# Inflammatory Response to Nano- and Microstructured Hydroxyapatite

**DOI:** 10.1371/journal.pone.0120381

**Published:** 2015-04-02

**Authors:** Gemma Mestres, Montserrat Espanol, Wei Xia, Cecilia Persson, Maria-Pau Ginebra, Marjam Karlsson Ott

**Affiliations:** 1 Materials in Medicine, Div. of Applied Materials Science, Dpt. Engineering Sciences, Uppsala University, Uppsala, Sweden; 2 Biomaterials, Biomechanics and Tissue Engineering, Dpt. Materials Science and Metallurgy, Technical University of Catalonia, Barcelona, Spain; University of California Merced, UNITED STATES

## Abstract

The proliferation and activation of leukocytes upon contact with a biomaterial play a crucial role in the degree of inflammatory response, which may then determine the clinical failure or success of an implanted biomaterial. The aim of this study was to evaluate whether nano- and microstructured biomimetic hydroxyapatite substrates can influence the growth and activation of macrophage-like cells. Hydroxyapatite substrates with different crystal morphologies consisting of an entangled network of plate-like and needle-like crystals were evaluated. Macrophage proliferation was evaluated on the material surface (direct contact) and also in extracts i.e. media modified by the material (indirect contact). Additionally, the effect of supplementing the extracts with calcium ions and/or proteins was investigated. Macrophage activation on the substrates was evaluated by quantifying the release of reactive oxygen species and by morphological observations. The results showed that differences in the substrate’s microstructure play a major role in the activation of macrophages as there was a higher release of reactive oxygen species after culturing the macrophages on plate-like crystals substrates compared to the almost non-existent release on needle-like substrates. However, the difference in macrophage proliferation was ascribed to different ionic exchanges and protein adsorption/retention from the substrates rather than to the texture of materials.

## INTRODUCTION

When a biomaterial is implanted into the body, a cascade of host reactions, including acute inflammation, wound healing and foreign body response, occurs at the tissue-material interface [1,2]. Besides isolating an infection or limiting the effects of trauma [3], acute inflammation is essential for promoting wound healing and restoring homeostasis [1,3,4]. Leukocytes are important mediators of acute inflammation and their activity may be moderated by both the chemical and physical properties of the biomaterial [4]. In the initial phase of inflammation, leukocytes (i.e. neutrophils and monocytes) are recruited to the site of injury where monocytes may differentiate into macrophages. Leukocytes respond to exogenous stimuli by releasing molecules such as reactive oxygen species (ROS) to destroy any pathogen or foreign body [1]. These cells may also release cyto- and chemokines, which can recruit additional immune cells to the damaged site and stimulate endothelial cells, fibroblasts, chondrocytes and mesenchymal stem cells to make new tissue [3,5].

The surface texture of biomaterials is believed to play a critical role in the cross-talk between cells such as inflammatory and mesenchymal stems cells. Various works have demonstrated that surface texture is capable of stimulating osteoinduction (de novo bone formation) by the recruitment and differentiation of mesenchymal stems cells to bone-forming osteoblasts [6–8]. The constant presence of macrophage-like cells during osteoinduction is believed to have an active role in recruiting osteoprogenitor cells through the release of chemical factors during inflammation [9,10]. As cell behaviour can be regulated through textural cues, the investigation of the inflammatory response due to different surface topographies is crucial to the design of biomaterials for improved bone regeneration.

Despite the important effect that texture of a bulk material has on inflammation, especially in the early events of bone healing, there have been very few studies done on this topic. Isolating the effects of texture is not easy as modifications of the material may also change its reactivity. This is the case for bioactive materials such as biomimetic hydroxyapatite (HA) [7]. HA, a widely accepted bone replacement material owing to its close similarity to bone’s mineral phase, reacts with the biological milieu through dissolution/precipitation processes which can lead to the formation of an apatitic bone-like layer. These reactions, aside from modifying the concentration of ions in the local biological milieu, might lead to the entrapment and adsorption of proteins on the materials surface. The local changes in ion concentration and the presence of an adsorbed protein layer are well known to influence cell behaviour [7].

HA substrates with controlled nano- and microstructures can be obtained through a cementitious reaction involving the hydrolysis of alpha-tricalcium phosphate [11]. Previous studies concerning the inflammatory response due to calcium phosphates have mostly focused on suspensions of nano- and microparticles with different characteristics (e.g. composition, size, shape, and sintering temperature) [12–17] or functionalized with peptides [12]. However, unlike these previous studies, the focus of the current experiments was to evaluate how nano- or microstructural features on calcium phosphate substrates can induce different inflammatory responses. To the best of our knowledge, such studies have not previously been done.

The goal of this article is to investigate the inflammatory response, in terms of macrophage proliferation and activation, of two HA substrates consisting of a network of needle-like or plate-like crystals, also taking into account the ionic exchanges inherent to the chemical properties of the material. The ultimate goal is to understand how texture (nano/microstructure) influences the inflammatory response and gain insight into how to better design surfaces for improved clinical performance of HA materials.

## MATERIALS AND METHODS

### 1. Preparation of material

Hydroxyapatite (HA) substrates with different textures were prepared through a cementitious reaction involving the hydrolysis of α-tricalcium phosphate (α-TCP). α -TCP was obtained by mixing calcium hydrogen phosphate (CaHPO_4_, Sigma Aldrich, ref. n. C7263, St. Louis, MO, USA) and calcium carbonate (CaCO_3_, Sigma Aldrich, ref. n. C4830) at a Ca/P ratio of 1.5. The powder mixture was subsequently heated in a furnace (Hobersal, Caldes de Montbui, Spain) in air at 1400°C for 15h, and finally quenched in air.

α-TCP was milled using an orbital miller (Pulverisette 6, Fritsch GmbB, Idar-Oberstein, Germany) and an agate jar and balls in order to produce two powders with different particle size distribution. Coarse powder (C) was prepared by milling 150 g of α-TCP with 10 agate balls (ɸ = 30mm) for 15 min at 450 rpm. Fine powder (F) was obtained performing a more energetic milling, first with 10 agate balls (ɸ = 30mm) for 60 min at 450 rpm and afterwards for 40 min at 500 rpm, and finally with 100 agate balls (ɸ = 10 mm) for 60 min at 500 rpm. Moreover, 2 wt% of precipitated hydroxyapatite (Merck, ref. n. 1.02143, Darmstadt, Germany) was added as a seed in the α-TCP powder.

Different HA substrates were prepared by mixing the C and F α-TCP powders with an aqueous liquid phase (2.5 wt% Na_2_HPO_4_) in a liquid to powder ratio of 0.65 ml/g. The formed paste was moulded in Teflon moulds of larger size for cell proliferation studies (ɸ = 15mm, h = 2 mm) and a smaller size to evaluate the release of ROS (ɸ = 6 mm, h = 2 mm). The discs were immersed in NaCl solution (0.9 wt %) until completion of the hydrolysis reaction (10 days) and afterwards rinsed with milliQ water (Reaction 1). Finally, the discs were dried at 37°C overnight. Hereafter, the nomenclature of the HA substrates prepared using coarse and fine α-TCP powder will be C-HA and F-HA, respectively.

$$3{Ca}_{3}{\left(PO_{4}\right)}_{2}+H_{2}O\;\to \;{Ca}_{9}(HPO_{4}){\left(PO_{4}\right)}_{5}(OH)$$ (Reaction 1)

The particle size distribution of the powders was analysed by laser diffraction (LS 13 320, Beckman Coulter, Bromma, Sweden). To minimize aggregation during measurement, the samples were dispersed in ethanol in an ultrasonic bath for 5 min. The specific surface area (SSA) of the powders and HA were determined by nitrogen adsorption using the Brunnauer–Emmet–Teller method (BET, ASAP2020 Micromeritics, Norcross, GA, USA). The morphology of the HA was evaluated with scanning electron microscopy (SEM, Zeiss Leo 1550, Jena, Germany).

### 2. In vitro studies

The HA samples were sterilized prior to *in vitro* studies by immersion in 70% isopropanol for 2 h and rinsed with milliQ water five times (5x 10 min agitation).

The *in vitro* study was divided into two parts. In the first part, proliferation of macrophages was evaluated in two different ways: cells were either seeded on HA surfaces (direct contact) or they were seeded on tissue culture polystyrene (TCPS) and cultured in HA extracts, i.e. medium previously incubated with HA (indirect contact). These two procedures were performed with the aim to determine whether the key parameter affecting cell growth was the material’s texture or the changes to the media caused by the substrates. In the second part, the activation of macrophages was determined after contact with different substrates by measuring the release of reactive oxygen species (ROS).

The *in vitro* studies were performed with a mouse leukaemic macrophage cell line (Raw 264.7). The cells were maintained in cell culture flasks in an incubator with a humidified atmosphere of 5% CO_2_ in air at 37°C. DMEM/F-12 medium (Thermo Scientific HyClone, ref. n. SH300023.01, Logan, UT, USA) supplemented with 10% fetal bovine serum (Thermo Scientific Hyclone, ref. n. SV30160.0) and 1% penicillin/streptomycin (Thermo Scientific Hyclone, ref. n. SV30010) was used for cell feeding. The medium was exchanged every other day. The cells were used for the experiments upon 80% confluence. Prior to plating, Raw 264.7 cells were detached by scraping in a single direction using a cell scraper (MidSci, ref. n. 99003, St. Louis, MO, USA).

#### 2.1 Cell proliferation on hydroxyapatite substrates

The proliferation of Raw 264.7 cells was evaluated by seeding 6·10^4^ cells on HA discs (3.4·10^4^ cells/cm^2^) placed in 24-well plates. As a control, cells were seeded on TCPS. Medium (1 ml) was changed daily. After 1, 3, 7 and 14 days, cells were washed twice with PBS and lysed for 50 min at 37°C with 500 μl of 0.1% Triton^®^ X-100 (Merck, ref. n. 1.08603.1000) in PBS. The number of cells was quantified by measuring lactate dehydrogenase (LDH) (Sigma-Aldrich, TOx7–1KT 091M6098). For this purpose, 50 μl was transferred to a 96-well plate together with 100 μl of LDH reagents (prepared by mixing equal amounts of substrate solution, cofactor preparation and dye solution). After incubating at room temperature for 20 min, the reaction was stopped with 15 μl of 1M HCl. The absorbance was measured at 490 nm with a background absorbance of 690 nm. The absorbance values were transformed to cell number by using a standard curve. The complete study was performed three times using quadruplicate samples.

Cell quantification was correlated with images of fluorescently stained cells on HA substrates. Live (green) and dead (red) cells were visualised using calcein/propidium iodide (Fluka, ref. n. 04511–1KT-F, St. Louis, MO, USA). Cells on HA discs and controls (TCPS) were rinsed twice with PBS and incubated for 15 min at 37°C with 0.067% calcein to stain live cells and 0.033% propidium iodide in PBS to stain dead cells. The cells were observed using a fluorescent microscope (Nikon Eclipse TE 2000-U, Melville, NY, USA), and images were taken using an imaging software (NIS-Elements F 3.0). Calcein was excited at 495 nm and propidium iodide at 536 nm. Green and red cells were quantified by means of ImageJ 1.46r (National Institutes of Health, USA). The image analysis was performed in parallel to the proliferation study and was repeated in three independent experiments.

Cell morphology on the different substrates was evaluated with SEM. The discs were soaked in 2.5% gluteraldehyde (SERVA, ref. n. 23115, Heidelberg, Germany) for 1 week. Afterwards, they were consecutively dehydrated in ethanol (10, 30, 50, 70, 90, 99.9%) for 10 min per dilution, being soaked in the first and last solutions twice. The discs were finally soaked in hexamethyl disilazane (HDMS, Sigma Aldrich, ref. 440191) solutions (1HDMS:2 ethanol, 1HDMS:1ethanol and 100% HDMS) for 20 min per dilution. The last solution was allowed to evaporate overnight. Finally, the samples were sputtered with a mixture of gold and palladium prior to visualization.

#### 2.2 Cell proliferation using extracts

HA extracts were prepared by soaking the HA discs (ɸ = 15 mm, h = 2 mm) in 1 ml of fresh medium in 24-well plates. Every 24 h, the media was replaced and the extracts were added to the cell culture. Two separate experiments were performed with the extracts. The first experiment aimed to evaluate the effect of extracts obtained from C-HA and F-HA on the cells. In the second experiment, we investigated the effect of supplementing the extracts with calcium and/or proteins (specifically, fetal bovine serum, FBS) before adding it to the cells.

*a) Indirect cell culture using extracts*. This experiment was performed using complete medium (cM, medium supplemented with 10% FBS and 1% penicillin/streptomycin) to prepare the extracts and fresh cM as control. 2·10^4^ Raw 264.7 cells were seeded in 96-well plates (6.3·10^4^ cells/cm^2^). The media (200 μl) was replaced by extracts after 4h (time 0). This procedure was repeated daily and cell number was quantified after 1, 3, 7 and 14 days. At each time point, the attached cells were lysed and lactate dehydrogenase (LDH) was quantified as previously described. The experiment was performed three times using quadruplicate samples.

In order to relate cell behaviour to changes in the media, the concentration of calcium and phosphorus ([Ca], [P]), pH and total protein concentration were measured. Ionic concentrations were determined by inducted coupled plasma—atomic emission spectroscopy (ICP-AES, Spectro Analytical Instruments, Kleve, Germany), measuring atomic calcium at 317.933 nm and phosphorus at 177.495 nm. [Ca] and [P] of extracts (diluted 10-fold with milliQ water) were measured in triplicate. The pH was monitored with a pH meter (Mettler Toledo S220 SevenCompact, Fisher Scientific, UK) using duplicate samples and total protein concentration was quantified using a Micro BCA^TM^ protein assay kit (Thermo Scientific, ref. n. 23235, Logan, UT, USA) following the manufacturer’s instructions. Extracts used for Micro BCA were prepared using phenol red-free MEM supplemented with 10% FBS and 1% penicillin/streptomycin (Gibco, ref. n. 51200–046, Carlsbad, CA, USA) to avoid any interference in the absorbance measurement. Extracts were taken from four samples and measured in sextuplicates (n = 24).

**Fig 1:**
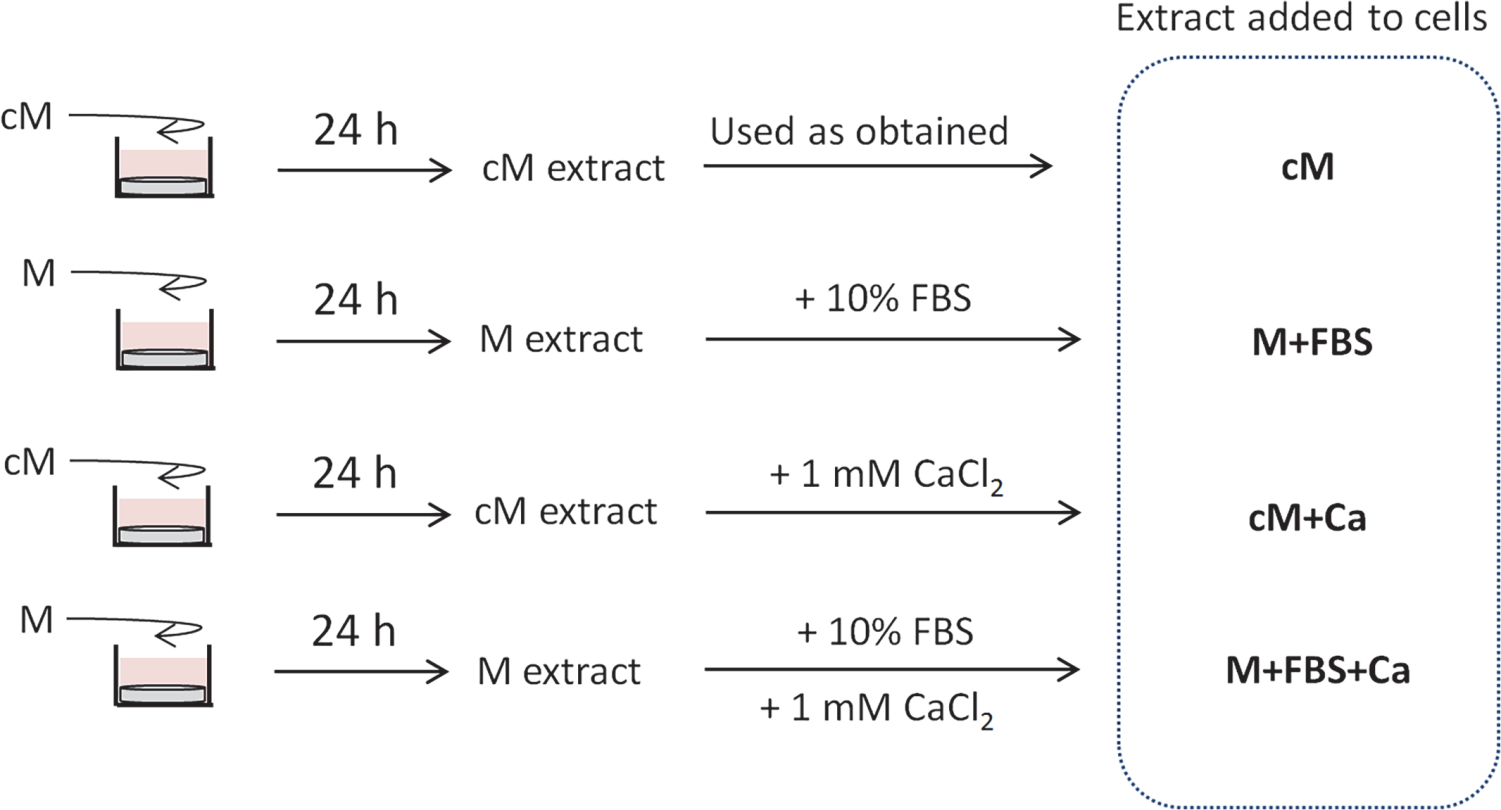
Schema of the preparation of extracts. Schema of the preparation of substrate extracts, which were either used as obtained or supplemented with Ca and/or fetal bovine serum (FBS). “M” stands for medium only supplemented with 1% penicillin/streptomycin. “cM” stands for complete media (DMEM medium supplemented with 10% FBS and 1% penicillin/streptomycin).

*b) Indirect cell culture using extracts supplemented with Ca and FBS*. HA is known to interact with the surrounding media by taking up proteins [18] and ions such as Ca [19]. To determine the effect of medium impoverishment on cell growth, extracts (medium conditioned with substrates for 24 h) were evaluated as obtained or supplemented with Ca, FBS or both. Cell proliferation/activation studies were essentially carried out as described above, however in this case, only 6.5·10^3^ cells were plated in each well (96-well plate; 2.0·10^4^ cells/cm^2^). Moreover, the culture medium used to soak the substrates contained either 10% FBS and 1% penicillin/streptomycin (complete medium, cM) or only 1% penicillin/streptomycin (medium, M). The extracts were collected every 24 h and either added as obtained or supplemented with 10% FBS and/or 1 mM calcium chloride anhydrous (stocks of 100% FBS and 100 mM CaCl_2_) to the cells. As control, fresh medium supplemented with 10% FBS and 1% penicillin/streptomycin was used. All media combinations are schematically explained in Fig. 1. Cells were grown in the established conditions with daily changing of the extracts. Cell number was quantified by LDH after 1, 3 and 7 days, as described previously. The complete study was repeated three times using quadruplicates.

#### 2.3 Release of ROS by macrophages

Activation in the form of total (intracellular and extracellular) ROS released by macrophages on HA substrates was quantified by a luminol amplified chemiluminescence assay [20]. Briefly, 100 μl of 2·10^6^ non-activated cells/ml (suspended in 4PBS/1DMEM/100 mM glucose solution) were seeded on the substrates (ɸ = 6 mm, h = 2 mm) pre-soaked in PBS and placed in white opaque 96-well plates. Immediately thereafter, 100 μl of luminol solution was added to each well. The luminol solution (500 μM) was prepared by adding 1% of luminol stock solution and 0.2% HRP (1 mg/ml) (Jackson Immuno Research, ref. n. 016-030-084, West Grove, PA) to a 4PBS/1DMEM/100 mM glucose solution. Luminol stock solution of 50 mM was prepared by dissolving the luminol (3-aminophthalhydrazide, ref. n. A/3150/44, Fisher Scientific, UK) in 0.2 M NaOH. Sample preparation was done in a dark room. Positive and negative controls were included by adding activated and non-activated cells directly to the well plate. As a positive control, macrophages were activated by adding phorbol-12-myristate-13-acetate (PMA, Sigma Aldrich, ref. n. P1585, 1 μM), a NADPH-oxidase activator [21]. Luminiscence was monitored in a microplate reader (Infinite M200, Tekan, Männedorf, Switzerland) at 37°C every two minutes for 60 min, using an integration time of 1000 ms and a settle time of 150 ms. Triplicate samples were used and the experiment was performed five times.

The number of cells attached on the substrates was evaluated after 1 h by the LDH assay, as previously described. In parallel, cells on other substrates were fluorescently stained with live/dead dye and quantified by means of ImageJ. [Ca] and [P] in extracts prepared in the same conditions (HA discs immersed in a 4PBS/1DMEM/100 mM glucose solution for 1 h) were evaluated by means of ICP-AES as previously described. Triplicates of each sample were analysed.

### 3. Statistical analysis

One way ANOVA was performed using IBM SPSS Statistics 19 software (IBM, Chicago, IL, USA at a significance level of α = 0.05. Scheffe’s post-hoc test was used in the case of homogeneity of variances (Levene’s test); otherwise, Tamhane’s post-hoc test was chosen.

## RESULTS

### 1. Material characterisation

**Fig 2:**
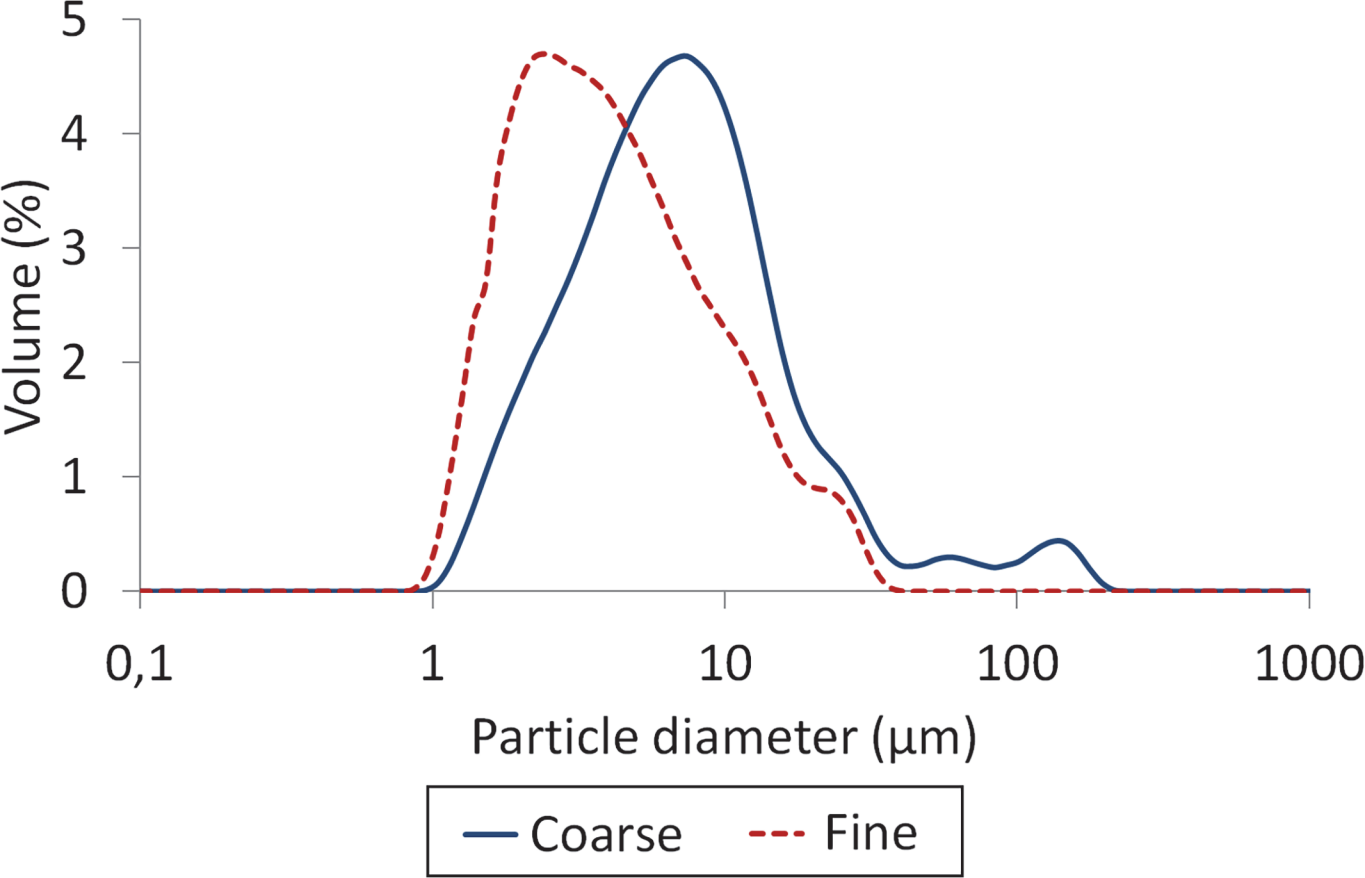
Particle size distribution of the cement powder. Particle size distribution of coarse and fine powder as determined by laser diffraction.

The milled α-TCP powder resulted in coarse and fine powder with a median particle diameter of 4.7 μm and 2.3 μm, respectively (Fig. 2). The SSA of the coarse and fine powder was 2.09 m^2^/g and 3.67 m^2^/g, respectively.

**Fig 3:**
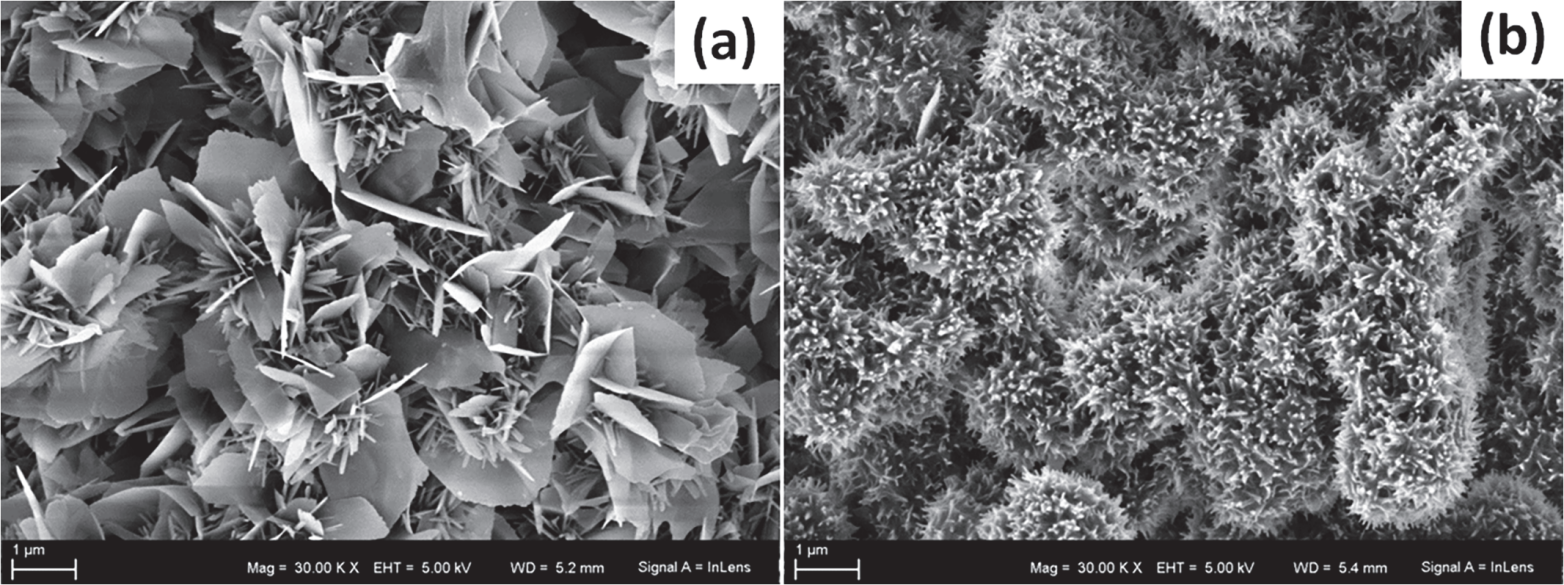
SEM of the substrates. Representative SEM micrographs of a) C-HA and b) F-HA prepared with a liquid to powder ratio of 0.65 ml/g.

The particle size of the α-TCP powder had a direct impact on the microstructure of HA substrates after setting (Fig. 3). As observed under SEM, C-HA and F-HA substrates were constituted by agglomerates of precipitated crystals. These F-HA agglomerates were smaller compared to C-HA. C-HA crystals had a plate-like morphology (Fig. 3A) whereas those of F-HA formed more needle-like structures (Fig. 3B). The microstructure of the samples also affected their SSA, which was 19.85 and 41.72 m^2^/g for C-HA and F-HA, respectively.

### 2. Cell proliferation on the hydroxyapatite substrates

**Fig 4:**
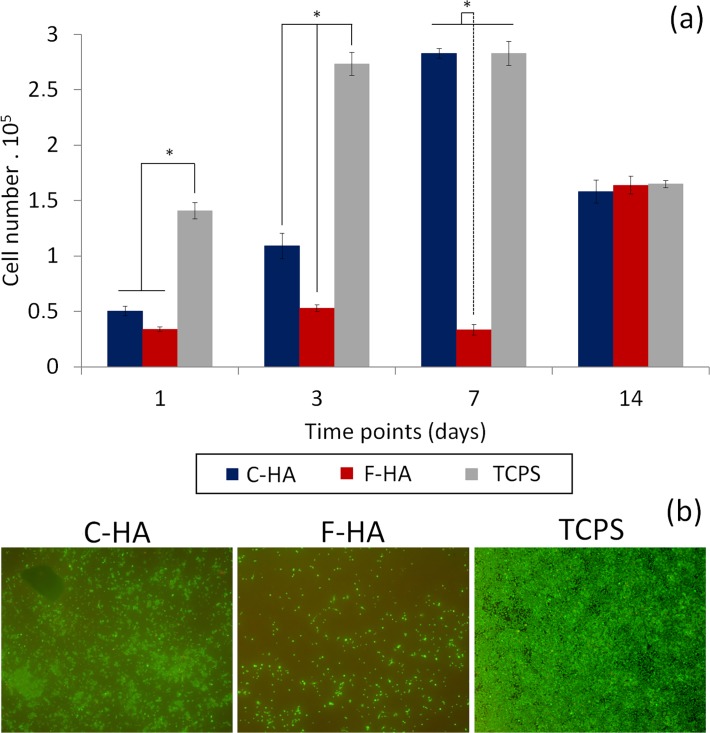
Direct contact assay. a) Proliferation of Raw 264.7 cells on substrate surfaces (C-HA and F-HA) and on tissue culture polystyrene (TCPS) (direct contact assay). 6·10^4^ cells were seeded on each sample. Cell number was quantified by LDH released after cell lysis (n = 4); b) Overlapping images of live/dead staining of cells on substrates at day 3. * indicates p < 0.05.

C-HA and F-HA were shown to affect cell proliferation differently over time (Fig. 4A). At day 1, the number of cells on TCPS was 3–4 times higher than that of HA substrates (p < 0.05), and cell growth on C-HA was slightly higher than that of F-HA (p > 0.05). At day 3, cells adhering to TCPS reached a maximum value, which was maintained for 7 days. In contrast, the number of cells on C-HA steadily increased for the initial 7 days. At day 14, a significant decrease in cell number (p < 0.05) was observed for both TCPS and C-HA. Cell growth on F-HA was significantly slower than on the other samples, and at day 7 the cell number on F-HA was significantly lower than either TCPS or C-HA (p < 0.05). F-HA only showed a significant increase in cell number at day 14 (p < 0.05). At day 14, the cell number on all three samples was similar (p > 0.05).

Live/dead cells were visualized with a fluorescent microscope after staining with calcein/propidium dye. Fig. 4B shows overlapping images of the live cells (green) and dead cells (red) at day 3. The number of live cells on C-HA and F-HA were 37.1% and 12.3% of that on TCPS, respectively, which corresponds to a C-HA/F-HA ratio of 3:1. The dead cells were only 1–3% of the total number of cells in each substrate, most likely due to the fact that they detached and were washed away during the course of the experiment. As for cell number, the visual results correlated well with the biochemical ones (LDH assay).

**Fig 5:**
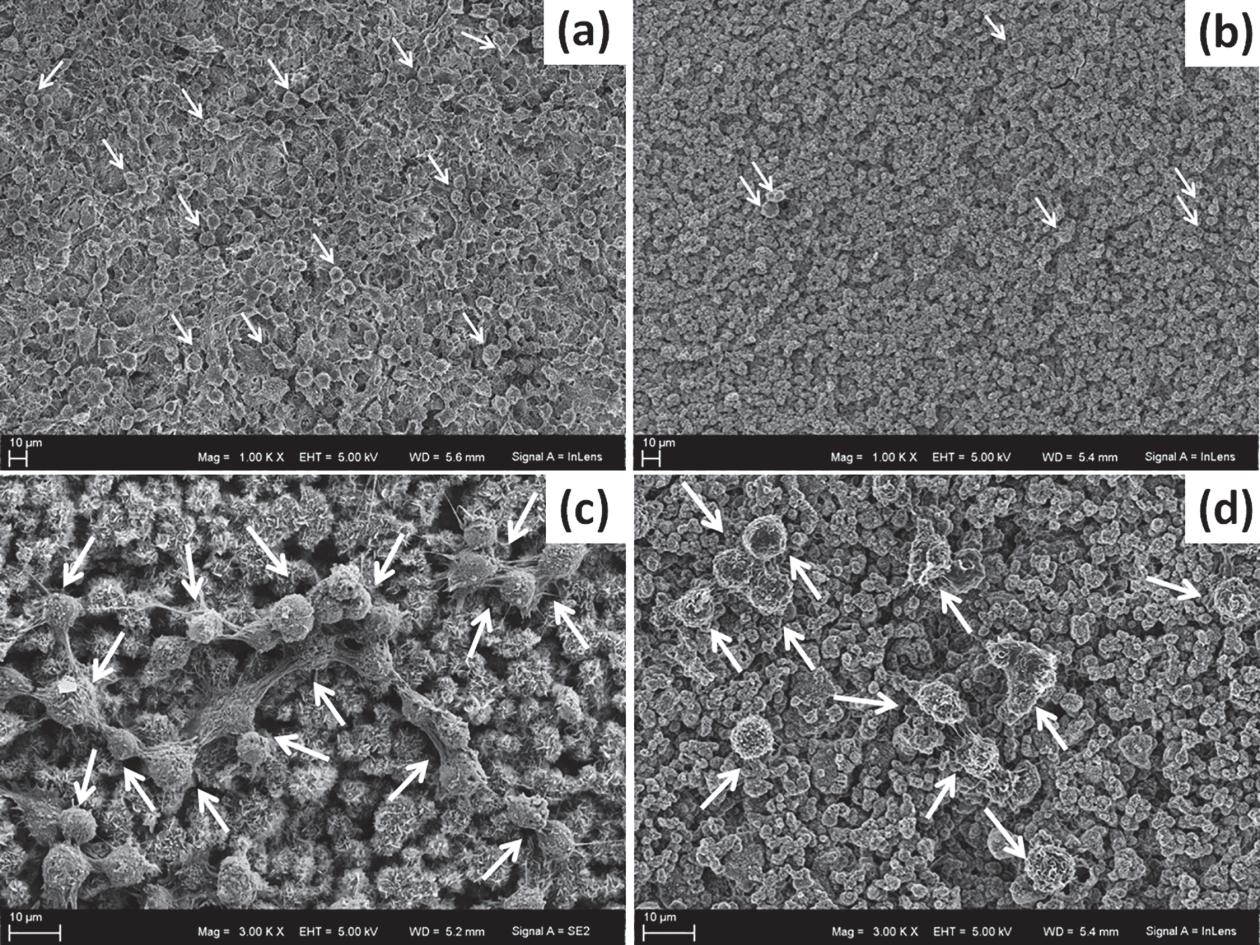
SEM of cells on substrates. Representative SEM micrographs of cells incubated on substrates for 3 days, a) C-HA and b) F-HA at low magnification (1000x); c) C-HA and b) F-HA at higher magnification (3000x). Arrows pointing on some of the cells.

Macrophage morphology on the HA discs was assessed at day 3 using SEM. Low magnification micrographs clearly show C-HA surface being covered by a large number of cells (Fig. 5A), whereas only a few cells adhered to the F-HA (Fig. 5B). Moreover, a difference in morphology could also be seen at higher magnifications. Cells on C-HA substrates were more spread out compared to the rounder morphology of macrophages cultured on F-HA (Fig. 5D).

### 3. Cell proliferation in hydroxyapatite extracts

#### a) Indirect cell culture using extracts

**Fig 6:**
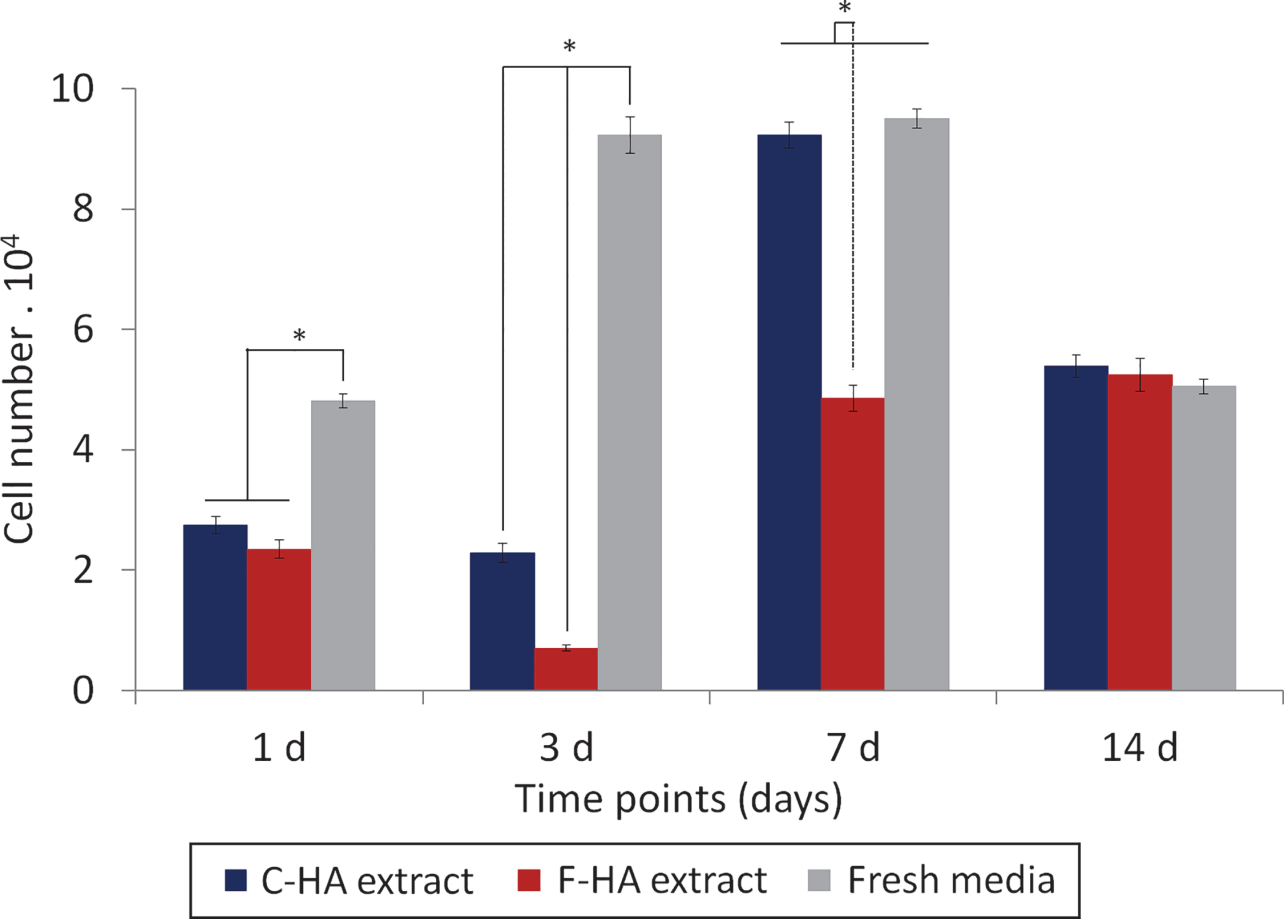
Indirect contact assay. Proliferation of Raw 264.7 cells in C-HA and F-HA extracts and in fresh media (indirect contact assay). 2·10^4^ cells were seeded to each well. Cell number was quantified by LDH released after cell lysis (n = 4). * indicates p < 0.05.

Fig. 6 shows the effect of cell proliferation using HA extracts as the medium being supplied to the cells (indirect method). At day 3, cell proliferation was only evident in the control wells using fresh medium, not for the cells cultured with extracts. The number of cells in fresh medium remained constant between day 3 and 7, whereas for both HA extracts, a prominent increase was observed. At day 14, a similar number of cells were present in all samples after a decrease in cell number for wells with C-HA extract and fresh medium.

**Fig 7:**
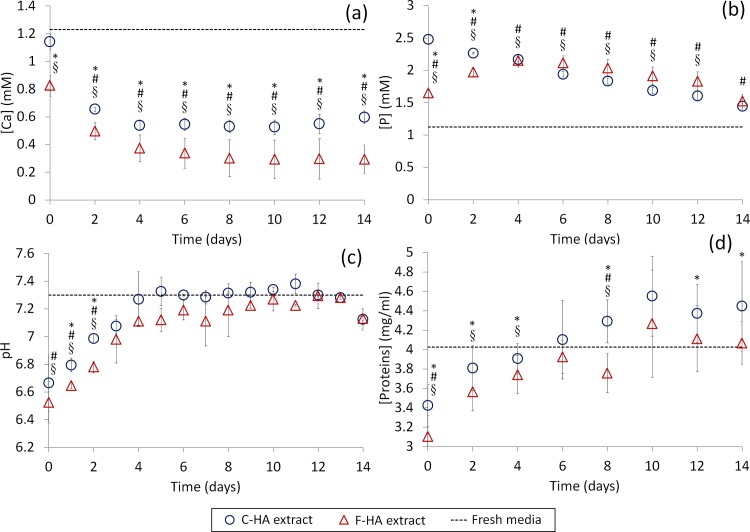
Characterization of the extracts. Characterisation of C-HA and F-HA extracts used for the indirect contact assay. Fresh media was used as control (dashed line). a) [Ca], b) [P], c) pH and d) total concentration of protein (fetal bovine serum, FBS). Extracts were prepared by soaking HA discs (ɸ = 15mm, h = 2 mm) in 1 ml of culture media (exchanged daily). Time 0 corresponds to extracts prepared 24 h prior to addition to the cells (n = 3 for a and b, n = 2 for c, n = 24 for d). * indicates p < 0.05 between C-HA and F-HA extracts; #indicates p < 0.05 between C-HA extract and fresh medium; § indicates p < 0.05 between F-HA extracts and fresh medium.

HA materials can interact differently with ions and proteins present in the media depending on their physico-chemical properties, i.e. chemistry, porosity and specific surface area [19]. [Ca], [P], pH and total amount of proteins in the HA extracts were determined (Fig. 7). Both HA materials take up a significant amount of Ca ions from the fresh media ([Ca] = 1.23 ± 0.02 mM) (p < 0.05), F-HA taking up a significantly higher amount than C-HA (p < 0.05) at every time point (Fig. 7A). After 4 days the amount of Ca uptaken by each HA was approximately constant, with a [Ca] of about 0.53 mM in the C-HA medium and 0.31 mM in the F-HA medium. Regarding [P], both C-HA and F-HA substrates released P ions into the media (original concentration, 1.12 ± 0.02 mM) and significantly increased (p < 0.05) the initial concentration of the media (Fig. 7B). For C-HA, the [P] decreased over time, whereas for F-HA, [P] peaked at 4 days and then decreased. Regardless of these differences, the [P] between the extracts was only significantly different (p < 0.05) at the initial time points. Both C-HA and F-HA decreased the pH of the media they were soaked in at short time points, this modification being significantly different for the initial 2 days (p < 0.05). At short time points, the medium containing F-HA was significantly more acidic than the one containing C-HA (p < 0.05 at 1 and 2 days) (Fig. 7C). The pH drop became less pronounced over time and returned close to the original value (7.31 ± 0.03) at day 4 for C-HA and at day 9 for F-HA. Finally, the total amount of protein in the extracts was measured with Micro BCA. At every time point, F-HA extract contained a lower amount of proteins than C-HA extract, this being statistically different (p < 0.05) at some of the time points (Fig. 7D). Interestingly, for the first 4 days, both extracts had lower protein content than fresh media. From day 5 onwards, the protein level recovered and no significant difference was observed between extracts and fresh media. No inorganic crystals were observed upon visual examination of the wells.

#### b) Indirect cell culture using extracts supplemented with Ca and FBS

**Fig 8:**
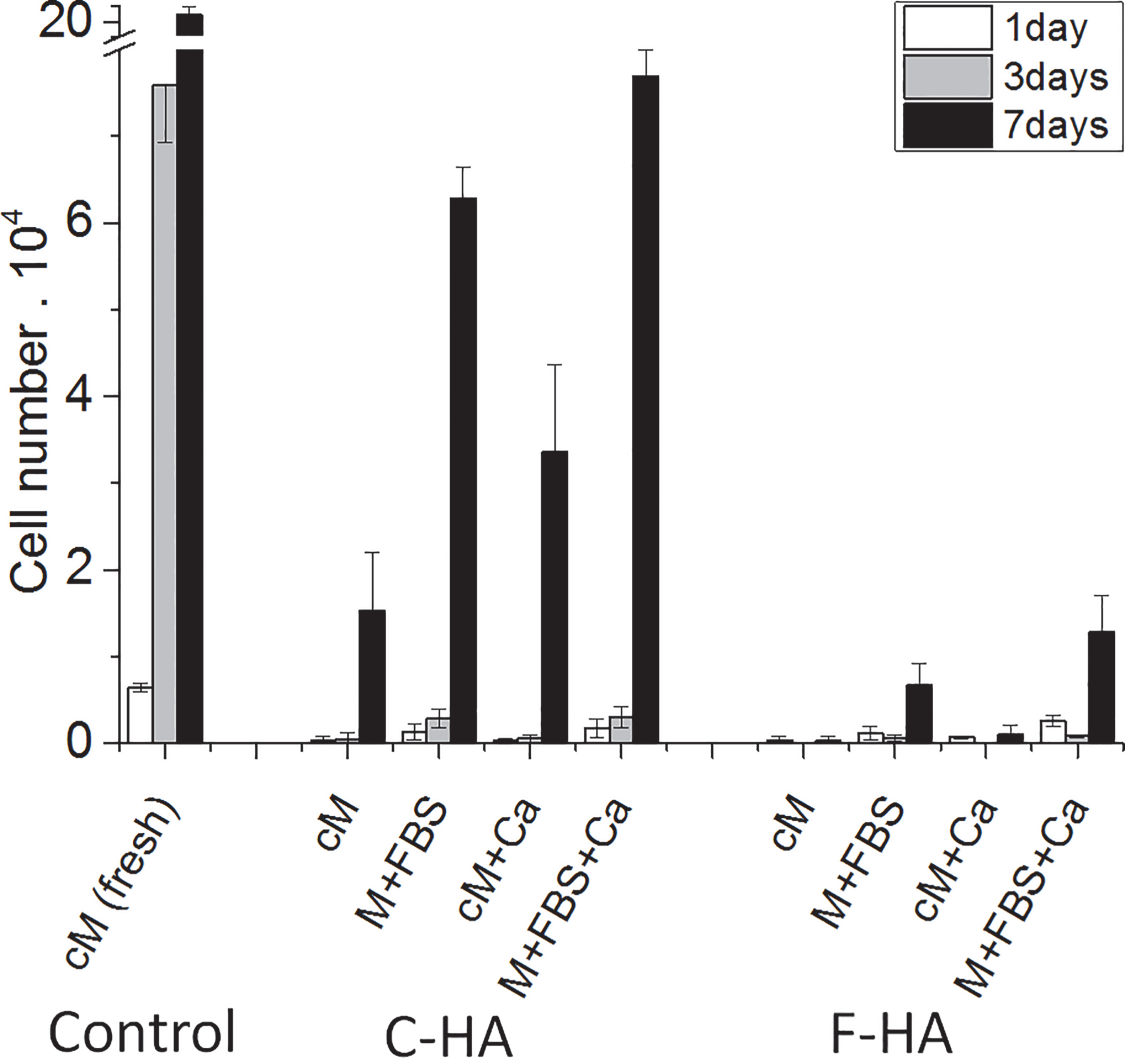
Indirect contact assay supplementing extract with Ca and/or FBS. Proliferation of Raw 264.7 cells (6.5·10^3^ cells per well) in C-HA and F-HA extracts supplemented with Ca and/or FBS. Four different extracts were used for each substrate. HA discs were soaked with complete media (cM, media supplemented with 10% FBS and 1% penicillin/streptomycin) that was either used after substrates soaking (cM) or was further supplemented with Ca (cM + Ca). Some other discs were soaked in media (M, containing no FBS) that was further supplemented with FBS (M + FBS) or supplemented with both FBS and Ca (M + FBS + Ca) (n = 4).

Fig. 8 shows the results of cell proliferation using fresh medium (control), HA extracts and HA extracts supplemented with Ca, FBS or both Ca and FBS. Interestingly, the addition of Ca and FBS enhanced cell proliferation for both C-HA and F-HA extracts, although significantly more for C-HA extracts. At day 7, cell proliferation was mostly enhanced by the addition of both Ca and FBS; and the addition of only FBS enhanced cell proliferation more than addition of only Ca. At all time points, fresh medium had significantly higher cell numbers than any other condition.

### 4. Activation of macrophages: release of ROS

**Fig 9:**
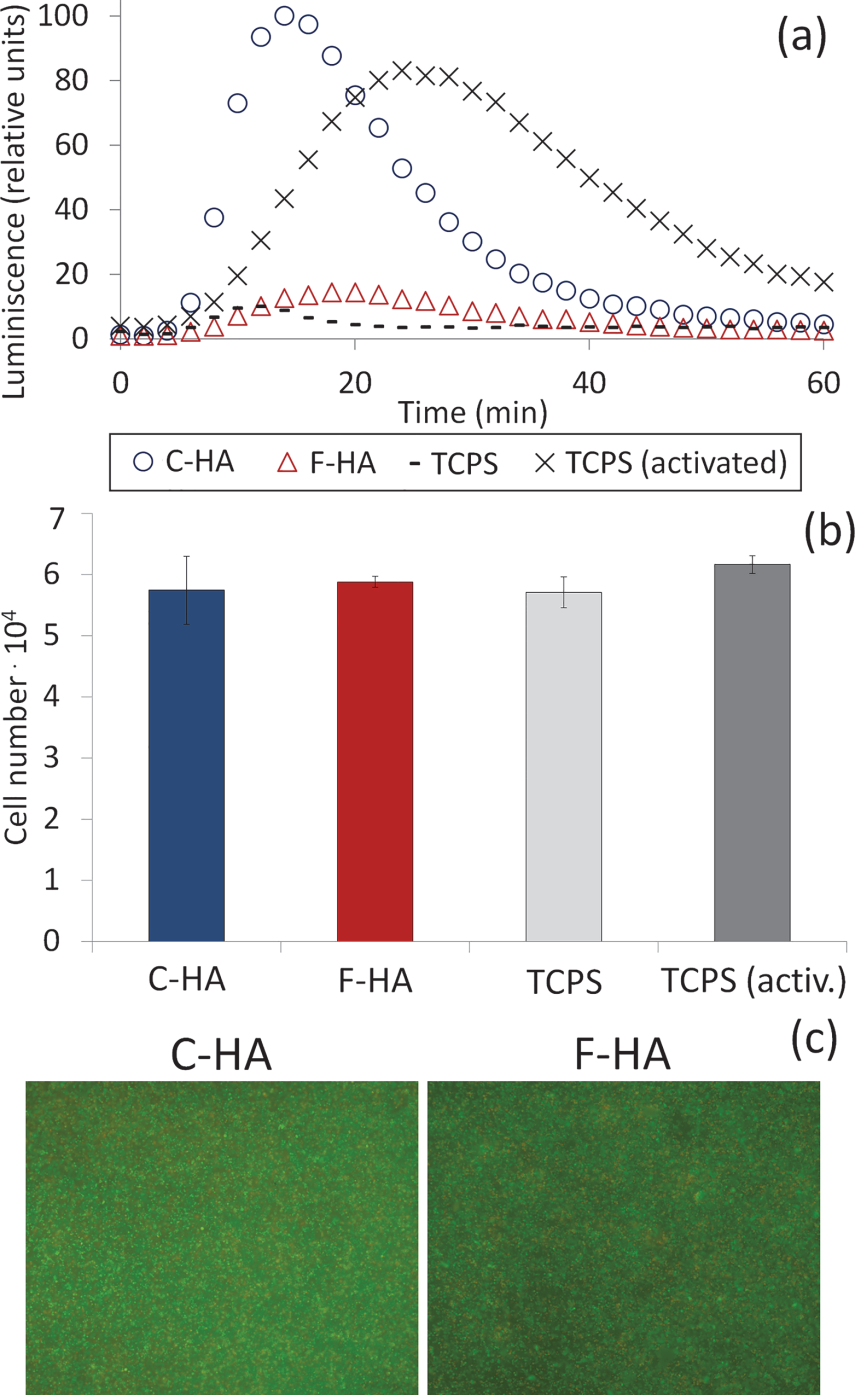
Release of Reactive Oxygen Species. Luminol-amplified luminescence assay (data normalized to relative units); b) Quantification of cell number after 1h, using LDH detection of lysed cells; c) Overlapping images of live/dead staining after 1h (n = 3).

A luminol-amplified luminescence assay was performed to evaluate the release of ROS from cells in contact with different HA substrates. ROS reacts with luminol and emits light, which can be kinetically monitored. The luminescence emitted is shown as relative light units obtained after normalization to the highest luminescence signal (Fig. 9A). Cells on TCPS produced ROS only when activated with PMA. This activation is shown by an intense and broad luminescence peak with a maximum around 25 min. A very weak signal was detected for non-activated cells, indicating absence or very low quantity of ROS. Interestingly, when non-activated cells were added to the topographically different substrates, unique luminescence patterns were observed for each sample type. A strong signal was only detected for cells in contact with C-HA, which was attributed to their release of ROS, whereas cells on F-HA produced a very weak signal, similar to that of non-activated cells on TCPS.

The number of cells adhered on the substrates at 1 h were quantified by LDH (Fig. 9B) and show no significant differences between substrates (p > 0.05). The quantification of green and red cells after live/dead staining (Fig. 9C) also indicated no differences between substrates (p > 0.05), with the presence of 65.7 ± 7.7% and 65.1 ± 5.3% live cells on C-HA and F-HA, respectively. The concentration of Ca and P measured in the media after the assay showed similar values regardless of the type of sample. These values did not differ by more than 11% when compared to the control (data not shown).

## DISCUSSION

**Fig 10:**
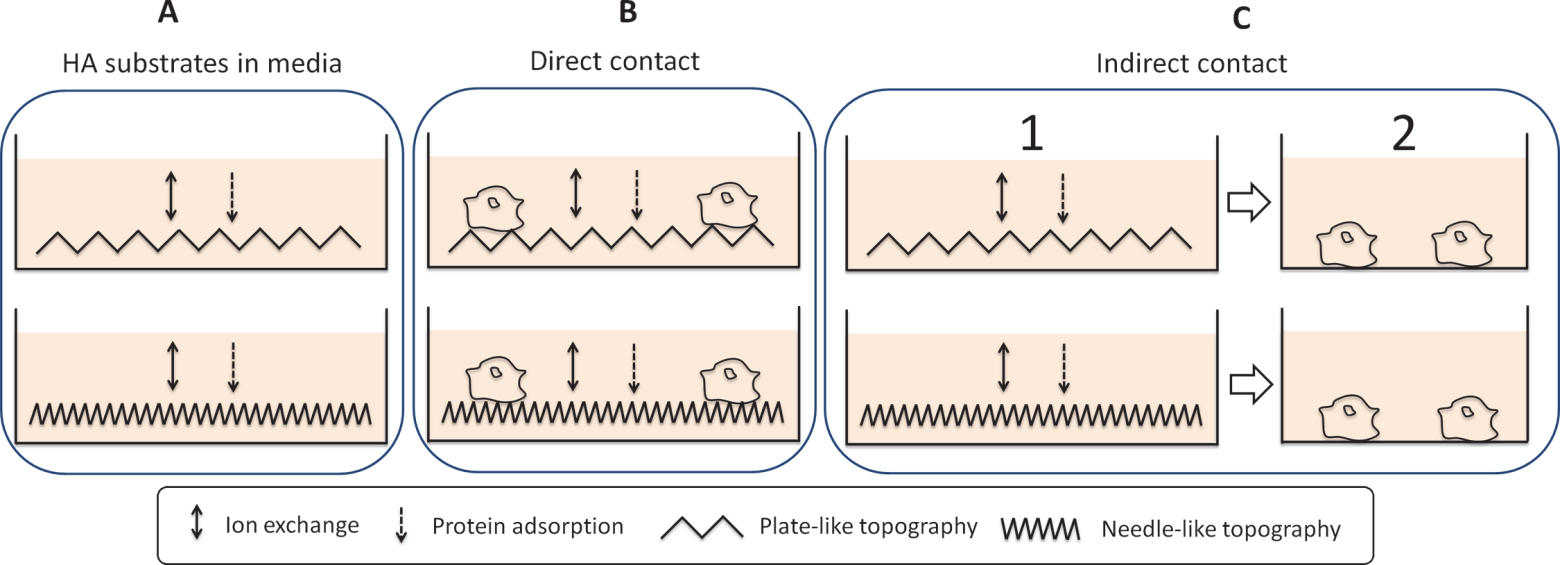
Factors involved in the experimental studies, where 1 represents the preparation of HA extracts and 2 the cell growth in these extracts.

The main motivation of this study was to better understand how nano and microstructured HA substrates affect the proliferation and activation of macrophages. Texture, as mentioned earlier, can be used to control cell behaviour and may be an important parameter in the design of biomaterials. Calcium phosphate cements have been used as a platform for the production of HA substrates with tailored textures. Since cementitious reactions occur through a dissolution and re-precipitation process, the materials obtained are porous due to the nature of the reaction, which results in an entangled network of crystals. This porosity implies that the bulk of the material interacts with the surrounding media (i.e. ions, proteins and other organic compounds) increasing the complexity of the system. Fig. 10 shows the factors involved in each of the studies performed.

The chemically identical HA substrates evaluated in this study were prepared using α-TCP with two different powder size distributions. When α-TCP powder is mixed with water, a dissolution-precipitation reaction starts from the surface of each particle, which acts as a template for the precipitation of calcium deficient HA (here labelled as HA for simplicity purposes) [11], producing agglomerates of crystals (Fig. 3). The crystal agglomerates produced in C-HA were bigger than those in F-HA due to the original particle size of the powder. The shape of the crystals grown on the surface of each particle is also dependent on the particle size of the initial powder [11]. The smaller particle size of the fine α-TCP powder, which had a higher SSA (3.67 m^2^/g), allowed a faster dissolution of the particles and thus a higher supersaturation of ions in the medium. Consequently, many nucleation sites appeared causing the precipitation of a high number of thin elongated crystals (needle-like) of HA that conferred the substrate with a SSA of 41.72 m^2^/g. In contrast, the dissolution of coarse α-TCP powder (2.09 m^2^/g) in the liquid phase was slower, producing fewer nucleation points. This resulted in plate-like crystals with a SSA of 19.85 m^2^/g.

The proliferation of macrophages on a biomaterial is particularly interesting to evaluate, as it could predict the severity of the inflammation upon implantation [22]. Firstly, macrophage proliferation was investigated directly seeding the cells on the different HA substrates including TCPS as control (Fig. 4). Macrophage proliferation followed the trend TCPS > C-HA > F-HA, however after 7 days the same number of cells was detected on both TCPS and C-HA. Whereas macrophages grew steadily on C-HA substrates from day 1, the growth was delayed on F-HA substrates and the most pronounced increase occurred between 7 to 14 days. This behaviour is in close agreement with a previous study performed with osteoblast-like cells [19]. The decrease in cell number at day 14 for both C-HA extract and TCPS could be ascribed to cells detaching due to high cell confluence i.e. the cell layer “peeling off”.

A priori, these differences could be related to the topographies of the substrates, indicating that plate-like crystals (C-HA) are more favourable for macrophage proliferation than needle-like crystals (F-HA) (Fig. 3). Similar results regarding the effects of topography were reported by Engel et al. for osteoblasts [19]. However, since HA is a bioactive material, its interaction with ions and organic molecules (e.g. proteins) present in the surrounding environment could also influence cell growth [23]. The ionic exchange is dependent on the chemical composition of both media [24] and material [25], as well as on the specific surface area of the material [19]. Moreover, the chemistry and texture of the HA substrates promotes the superficial adsorption and entrapment of different types of proteins [26] [27], which could play a major role in directing cellular interactions [28,29].

An indirect contact study was performed to isolate the influence of the microstructure versus that of the chemical/biological composition of the medium on cell proliferation. In the indirect study, HA extracts delayed the cell proliferation in comparison with the fresh media, and F-HA extracts delayed the cell growth more than C-HA extracts (Fig. 6), which was in agreement with the direct contact assay. Since the cell growth in the indirect contact was only dependent on the composition of the extracts, [Ca], [P], pH and protein concentration were monitored throughout the course of the experiment.

Fig. 7A showed uptake of Ca ions by both F-HA and C-HA throughout the experiment. The high capacity of the materials to adsorb Ca ions was hypothesized to be due to the large number of vacant sites in the crystal structure of the calcium deficient hydroxyapatite structure precipitated from the hydrolysis reaction of α-TCP [24]. Around 57% of Ca ions from media were adsorbed daily by the C-HA, a value close to that reported by Gustavsson et al. for a HA with a similar SSA [24]. Interestingly, the capacity of F-HA to adsorb Ca ions was higher (75%), which could be ascribed to its larger surface area. Both HA substrates released phosphates into the media over time (Fig. 7B). This release was slightly higher for F-HA, in accordance with previous work [24], probably due to its larger SSA. Since both HA adsorbed Ca ions and released P ions it was expected that the Ca/P ratio of the substrate surface would change. To preserve electroneutrality, the material would adsorb other anions such as carbonates or hydroxyls. In fact, a decrease in pH in the culture media was recorded at early times points (Fig. 7C), in agreement with Gustavsson et al. [30], which is consistent with the uptake of hydroxyls and release of protons (in addition to phosphates) [24]. A more prominent drop in pH was observed for the medium containing F-HA (p > 0.05) probably due to its higher SSA. With regards to protein adsorption, no significant differences were observed between the two substrates (Fig. 7D). Interestingly, an overall decrease in the protein content in the culture medium was recorded at early time points, suggesting protein adsorption/retention by the materials. Six days were needed to saturate the porous materials. The avidity of such substrates for proteins was earlier reported by Espanol et al. [18,26].

When comparing both, direct and indirect data (Figs. 4 and 6), we can see a rather similar proliferation trend, yet the individual contributions of texture and ionic environment are also visible. From the indirect culture experiment (setup where surface topography is not involved) it is clear that the depletion of ions/proteins is critical, as cell proliferation cannot reach the same level as in the control, this effect being more pronounced in the F-HA extracts, where the changes in ionic concentrations were higher. Moreover, since the direct culture experiment (cells exposed to materials with different surface topographies) showed a rather similar trend to that observed in the indirect culture, this finding further supported the fact that ionic/protein exchanges had a more dominant role than topography in affecting cell proliferation. However, the noticeable delay in cell proliferation observed for the cells cultured on the F-HA substrates, compared to the indirect assay, proves that topography still plays a role. In an attempt to further elucidate the role of ionic/protein exchanges, and due to the numerous studies supporting the importance of [Ca] and proteins for cell metabolism and growth [31–38], another series of indirect studies were performed. In this study, cells were cultured with HA extracts supplemented with either Ca, FBS or both, at a physiological level, before adding them to the cells.

The results of this modified indirect cell culture study showed that for both HA extracts, maximum cell growth occurred when both Ca and FBS were added, and supplementation with FBS enhanced cell proliferation more than Ca alone. This finding confirmed that the depletion of essential ions (e.g. Ca) and proteins in the media by the adsorption/retention on HA compromised cell growth. On one hand, Ca is an intracellular messenger that regulates a large number of cellular functions [39]. Yamuchi et al. showed that bone marrow mononuclear cells (J774 cell line) detect changes in extracellular [Ca] via their calcium-sensing receptor, thereby causing changes in cell migration and proliferation in response to this signal [34]. Collart et al. also proved that a medium free of Ca limits the transcription of the c-gene by macrophages, thereby affecting proliferation of these cells [35]. Similarly, Kaibuchi et al. concluded that a [Ca] higher than 0.5 μM is needed to enhance DNA synthesis and thus permit proliferation of macrophages [36]. Briefly, even small changes in extracellular [Ca] can alter metabolic functions. On the other hand, proteins contribute with vital nutrients, attachment factors and growth factors [40]. Therefore, cell viability and proliferation can be compromised if essential proteins are absent [38]. However, it was outside of the scope of the current study to determine such differences. Moreover, HA decreased the pH of the surrounding media (i.e. extracts) at early time points. Cell viability can also be affected by a decrease in the extracellular pH, including cytosolic and membrane associated enzyme activities, ion transport activity and synthesis of proteins and DNA [41].

The overall results indicated that changes in concentration of both ions and proteins, which were observed through the direct and indirect cell cultures (more pronounced for F-HA than for C-HA extracts), were the principal factors affecting cell proliferation. HA extracts delayed cell growth and this delay was more pronounced for F-HA substrates (Figs. 7 and 8). Although Figs. 7 and 8 showed the same trends, they should be compared with care as different cell number was seeded for each study. When comparing both substrates, the entangled network of needle-like crystals and the higher SSA of the F-HA caused a major drop of Ca and protein concentration as well as a lower pH value at early times, causing a more severe delay in cell growth. However, since the supplementation of both Ca and FBS to the HA extracts did not reach the same proliferation level observed in the control wells, (Fig. 8) it can be supposed that other inorganic or organic species also controlled cell proliferation. It was beyond the scope of this work to determine which ion(s)/molecule(s) was responsible for such behaviour.

In addition to studying macrophage proliferation on different HAs, macrophage activation was evaluated by measuring the amount of released ROS when in contact with the substrates [1,42]. A distinct release of ROS was seen in relation to the type of material that the cells were exposed to (Fig. 9A). C-HA caused a burst of ROS similar to that of activated macrophages. In contrast, F-HA induced a much smaller ROS release, similar to that of non-activated cells on TCPS. No difference in cell number was seen between the substrates in the course of this experiment (1h) as determined by LDH quantification (Fig. 9B) and live-dead staining (Fig. 9C). SEM investigations confirmed that cells on C-HA were more activated as indicated by their flattened morphology, (Fig. 5C), which is common for cells attempting to endocytose or degrade a foreign body [43]. In contrast the majority of cells on F-HA had a round morphology (Fig. 5D), indicating a lesser extent of activation.

The different level of macrophage activation on each substrate was mainly attributed to topographic differences rather than ionic exchanges with the surrounding media. The fact that quantification of ROS is a fast test lasting no more than 1h precludes a drastic change in the concentration of ionic species as observed from a variation of less than 11% in the concentration of Ca and P from the media (data not shown). Studies with titanium, poly-vinylidene fluoride and polytetrafluoroethylene had previously shown that microstructured patterns cause higher activation of inflammatory cells compared to nanopatterned surfaces [44–46]. Therefore, we hypothesize that the fact that macrophages were activated in contact with plate-like crystals of C-HA but not on needle-like crystals of F-HA could be ascribed to the different topographies of the materials. The plate-like crystals having a micrometric size, would thus trigger a higher activation response than the nanometric dimensions of the needle-like crystals. It is difficult to compare the results of the current study with other studies evaluating the inflammatory response of HA nanoparticles, since in most of these studies the inflammatory reaction was triggered when the particles were phagocytized by the cells.

In light of these results, a better clinical performance of F-HA could be expected due to their lower stimulation of inflammatory cells. However, as previously explained, activated inflammatory cells trigger the release of ROS as well as the release of cytokines, which in turn can up-regulate the osteogenic expression of cells such as mesenchymal stem cells [47]. From this perspective, it could be hypothesized that C-HA would induce bone healing in a higher degree than F-HA. In any case, the contrast in the level of macrophage activation by the texturally different HA substrates is of high interest, since it shows that the inflammatory response can be tuned by modifying an easily controllable parameter such as the powder particle size.

Overall, the present work shows that the textural differences of compositionally identical calcium phosphates indirectly affect macrophage proliferation through different ionic exchanges and protein adsorption capabilities. In contrast, the different degree of activation of the macrophages was directly related to the microstructure of the surfaces. To pinpoint the specific contribution of each of these factors in macrophage behaviour is not possible at present and would require the use of less complex materials to elucidate each factor. This study has brought us one step closer to understanding how texture influences the immune response of HA substrates, which is an important factor when designing surfaces for improved clinical performance.

## CONCLUSIONS

The present work has proved that hydroxyapatite substrates consisting of an entangled network of either micrometric plate-like crystals (C-HA) or nanometric needle-like crystals (F-HA) exhibit very different behaviours in terms of macrophage proliferation and activation. Cell proliferation was slower on F-HA than on C-HA for both direct and indirect contact. Although the microstructure likely played an important role, the delay in cell proliferation was mainly associated with a more pronounced depletion of ions (e.g. Ca) and proteins in the cell medium in contact with F-HA, likely due to its high specific surface area. In contrast, microstructures seemed to be the main cause for difference in macrophage activation between the two HAs, where cells on plate-like crystals released higher amounts of ROS and adopted a more activated morphology.
